# Investigation of Damage Reduction When Dry-Drilling Aramid Fiber-Reinforced Plastics Based on a Three-Point Step Drill

**DOI:** 10.3390/ma13235457

**Published:** 2020-11-30

**Authors:** Fu-Ji Wang, Meng Zhao, Jian-Bo Yan, Shen Qiu, Xin Liu, Bo-Yu Zhang

**Affiliations:** Key Laboratory for Precision and Non-Traditional Machining Technology of Ministry of Education, School of Mechanical Engineering, Dalian University of Technology, Dalian 116024, China; wfjsll@dlut.edu.cn (F.-J.W.); zmengsym@163.com (M.Z.); yanjianbo1216@163.com (J.-B.Y.); msts@mail.dlut.edu.cn (S.Q.); liuxin1997@mail.dlut.edu.cn (X.L.)

**Keywords:** AFRP, drilling, damage reduction, three-point step drill

## Abstract

Aramid fiber-reinforced plastic (AFRP) is widely used in bullet-proof and armor structures, and is difficult to drill because of the high-toughness aramid fibers with ductile fracturing—differently from carbon fiber. Therefore, drilling quality cannot be ensured by the drilling used for carbon fiber-reinforced plastic, and frequently, delamination and burrs occur in the drilling process. This article first established a two-dimensional cutting model for analyzing the fiber deformation and material interface cracking. According to the model, reducing the thrust force and the radial force of the edge on the fibers is an effective way to reduce the fiber deformation, and a three-point step drill is proposed further. Comparative experiments were carried out among twist drilling, candle core drilling and three-point step drilling under three drilling parameters. The results show that the three-point step drill changed the traditional cutting behavior on the drill-exit material into a compound process. Finally, the AFRP was cut effectively with the novel drill with a small thrust force, and the delamination and “burrs area” were reduced through different drilling parameters. In summary, the three-point step drill can drill the AFRP without delamination and burrs with 0.02 mm/rev, which provides a new solution of cost-effective production for AFRP manufacturers.

## 1. Introduction

Aramid fiber-reinforced plastic (AFRP) is made up of the aramid fibers with excellent toughness and plastic, after laminating in a certain orientation and curing under high temperature and pressure conditions. AFRPs have been widely used in aerospace, military and automotive industries due to their excellent toughness and impact resistance when receiving high dynamic and local impacts [1,2,3]. To date, AFRP is applied to all warplanes and to tank armor as the main structural material; in this market, China has reached 1.23 billion RMB and keeps up about 10% growth every year. Although AFRP components can be integrally formed in near net shape, thousands of holes are still necessary to be made for assembly within strict damage tolerance in the component manufacturing process [4,5]. At present, hole-making technology mainly includes drilling, water jet, laser and so on. Among them, dry-drilling in one-shot is still the most effective and far-ranging way for manufacturers to make holes currently [6,7,8].

As is known widely, the delamination and burrs occur often in the drilling process of fiber-reinforced plastics, such as carbon fiber-reinforced plastic (CFRP) and AFRP [9]. For the CFRP, Zhang et al. [10] predicted the cutting forces based on the orthogonal cutting model, and found that the high cutting force is the main reason for cracking of fiber/resin. Pwu and Hocheng [11] proposed a cutting model for the carbon fiber at the cutting angle of 90°according to the cantilever beam theory on fiber. As such, many orthogonal cutting models have been reported for analyzing the fiber/resin bending and exploring the low-damage cutting method [12,13], which has clarified the cutting damage formation of CFRP in orthogonal cutting. In addition, new drills were invented: Hao et al. [13] proposed a novel clip-edges drill structure which can cut the fibers from both sides and reduce fiber resin cracking and obtained more high-quality holes. Jia et al. [14] invented a novel intermittent-sawtooth drill structure, and the cutting lips of the structure could reverse the cutting direction from downward to upward, and thereby reduce the drill-exit damages. Fei Su et al. [15] compared the dagger drill and the brad-spur drill, and proposed a novel drill which reduced the thrust force and the damages upon drill-exit. Those previous studies showed that the drilling technology of CFRP has been developed in detail.

However, different characteristics for the AFRP and CFRP lead to the significant differences in drilling-damage mechanism. First, AFRP with lower interfacial bonding than CFRP leads to interface deboning being more likely to occur [16]. More importantly, the aramid fiber is a flexible material with high toughness, and carbon fiber has brittleness and hardness; they are completely different [17,18], which makes the cutting form and damage formation process of AFRP significantly different from CFRP’s [19], and the aramid fiber tends to slip along the cutting direction. As such, the previous studies mainly focused on the drilling process of CFRP, which is not suitable for the AFRP, and serious damages (delamination and burrs) are extremely common in the drilling of AFRP [20]. These damages greatly decrease the connection strength between components, which will reduce the life-span of the assemblies, endanger the safety of users and reduce the reliability of equipment [21,22,23]. Therefore, studying the drill-exit damage formation processes and proposing an approach to reduce the drilling damage to AFRP laminates are full of challenges.

Under the urgent needs of the aviation industry for high-quality AFRP drilling, drilling parameters and drilling tool structure are the main ways for reducing the drilling damages. In the case of drilling parameters of AFRP, A. N. Shuaib et al. [24] observed that the drill with high spindle speed could reduce the burrs and delamination damages in the drilling process for a drill bit with a 135°-point angle and TiN-coating. Meanwhile, F. Veniali et al. [25] also found that high spindle speed and low feed rate will reduce drilling torque and thrust force, thereby reducing AFRP drilling damages. The above research shows that lower feed per tooth (high spindle speed, low feed speed) is a way to improve the drilling quality of AFRP. However, in this way, the efficiency of drilling is greatly reduced, and according to the research of M Iqbal et al. [26], lower feed per tooth will greatly aggravate the tool to wear and make the drilling-life drop sharply [27]. As mentioned, the drilling parameters’ optimization cannot meet the drilling-quality requirements of AFRP application.

Then, for improving the drilling quality, due to the drill bit geometry having a significant effect on damages, efforts have been made toward drill structure. G.R. Bishop et al. [28] studied the influence of the point angle of the drill on quality and found that a point angle over 180°can reduce delamination damage. D. Bhattacharyya et al. [29] also found a twist drill with a 220°-point angle can obtain better drilling quality for AFRP. Jin Lei et al. [30] carried out the experiment using a candle core drill, an eight-sided drill and a twist drill for drilling AFRP, and the results showed that candle core drill can reduce the delamination and burrs for AFRP. The research of Anarghya A et al. [31] and A. Díaz-lvarez et al. [32] also found similar results. The candle core drill is generally considered as the preferred to drill AFRP. Additionally, Hang Gao et al. [33] proposed a new type of sawing drill which can reduce by 20–25% thrust force and drilling damages. Sinan Liu et al. [34] proposed that adding annulus support on the drill-exit side can improve the drilling quality of AFRP. The research on the above-mentioned guides the realization of AFRP low-damage drilling holes. However, most researchers focused on reducing the maximum thrust force, which hardly achieves a substantial improvement in burrs, and comparing the drills which have been applied to drilling metal and CFRP, which cannot realize drilling the AFRP with high-quality. Therefore, there is an urgent need to establish a theoretical model for the drill-exit damages, and further to develop a novel drill for minimizing the drilling damages on AFRP.

This study first established a two-dimensional drilling model on drill-exit of the fiber; the deformation of the fiber was theoretically calculated and analyzed; the sensitive factors affecting the fiber deformation were obtained. Then, based on the model, the drilling method for drill-exit with small fiber deflection was proposed to reduce drilling damages. Further, a novel three-point step drill was generated in theory, which is associated with the drilling model, and suitable drilling parameters were matched. Comparative drilling experiments are carried out and the drilling damage was reduced by the novel three-point step drill when dry-drilling AFRP.

## 2. Drilling Model of AFRP

### 2.1. Drill-Exit Damages Analysis

**Figure 1:**
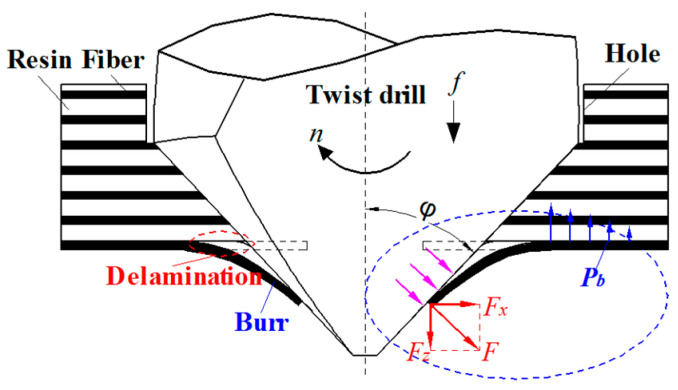
Damages production model in drilling AFRP.

As shown in Figure 1, when the high thrust force is greater than the bond strength in a drilling process, the fiber/resin bending will be caused, which will lead to delamination damage. Besides, aramid fiber with extremely high toughness which is being cut will easily produce downward bending deformation; then the fibers cannot be cut by the drill which lead to the burrs in drill-exit. According to the drilling process in Figure 1, the point angle of the drill was *φ*; the force of the drill on the drill-exit aramid fiber can be simplified as the force *F*, which can be illustrated as radial cutting force *F_x_* and thrust force *F_z_*. As to the fiber was bonded by the resin, the intensity of the bonding force was *P_b_* and the fiber plastic/polymer bonding strength was σ_b_ [35]. It is clear that the fiber deformation under the cutting force should be calculated for designing the novel AFRP drill to improve the drilling quality.

### 2.2. Fiber Deformation Calculation

**Figure 2:**
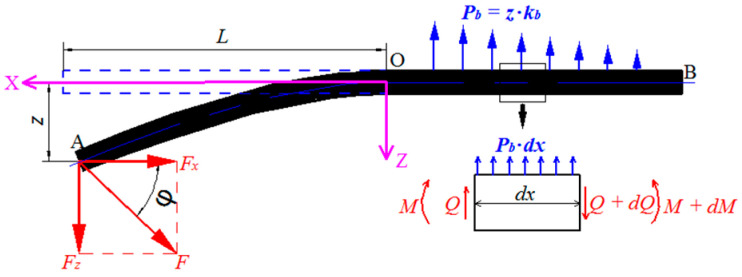
Two-dimensional beam model of the drill-exit fiber for AFRP.

As mentioned above, the fibers in drill-exit can be simplified to the beam model, as shown in Figure 2. For the convenience analysis, the additional x-z coordinate system is used for convenience and the coordinate origin O is the bonding point. The left and right side of the O can be regarded as the cantilever beam and the foundation beam, respectively. Point B is located at the limit where the drilling force is not affected and point A at the left end of the fiber.

For the case on right side of O, select the micro-body length *dx*; the equilibrium equation can be obtained as follows [12]:

(1) $$\left\{ \begin{array}{cc} \sum Z=0 & Q-(Q+dQ)+p_{b}dx=0\\ \sum M=0 & (M+dM)-M-(Q+dQ)dx-k_{b}\frac{dx^{2}}{2}=0 \end{array}\right.$$

where *Q* (*= −E_f_I_f_d^3^z/dx^3^*) represents the shear force; *E_f_* and *I_f_* are the elastic modulus and moment of inertia separately; *M* is the bending moment; and *k_b_* is the equivalent modulus of the bonding interface [28]. Then, according to the boundary conditions of the drilling process, the right-side fiber deformation *z*_1_ curve and deflection *θ*_1_ can be obtained as follows:

(2) $$\left\{ \begin{array}{l} z_{1}=e^{-\lambda x}\left[\frac{\sigma_{b}}{k_{b}}\cos\lambda x-\left(\frac{2\lambda \sigma_{b}}{k_{b}}+\frac{\sigma_{b}}{k_{b}}\right)\sin\lambda x\right]\\ \theta_{1}=-\lambda e^{-\lambda x}\left[\left(\frac{2\sigma_{b}}{k_{b}}+\frac{2\lambda \sigma_{b}}{k_{b}}\right)\cos\lambda x-\frac{2\lambda \sigma_{b}}{k_{b}}\sin\lambda x\right] \end{array}\right.$$

where $\lambda =\sqrt[{4}]{k_{b}/4E_{f}I_{f}}$; then, the deformation and deflection of fiber at point O are shown in Equation (3):

(3) $$\left\{ \begin{array}{l} {z_{1}|}_{x=0}=\frac{\sigma_{b}}{k_{b}}\\ {\theta_{1}|}_{x=0}=\frac{2\lambda \sigma_{b}}{k_{b}}+\frac{2\lambda^{2}\sigma_{b}}{k_{b}} \end{array}\right.$$

For the case of the right side of O, the equilibrium equation of fiber is shown in Equation (4):

(4) $$E_{f}I_{f}\frac{d^{2}z_{2}}{dx^{2}}=-F_{z}\cdot x-z_{2}\cdot F_{x}\;0\leq x\leq L$$

The left-side fiber deformation *z*_2_ curve is obtained by Equation (5):

(5) $$z_{2}=C_{1}\cos\sqrt{\frac{F_{x}}{E_{f}I_{f}}}x+C_{2}\sin\sqrt{\frac{F_{x}}{E_{f}I_{f}}}x-\frac{F_{z}}{F_{x}}x$$

where *C*_1_ and *C*_2_ as constants of integration are determined by the boundary conditions, and then according to the boundary conditions, the aforementioned parameters *C*_1_ and *C*_2_ are as follows:

(6) $$\left\{ \begin{array}{l} C_{1}=0\\ C_{2}=\frac{F_{z}}{F_{x}} \end{array}\right.$$

According to the displacement superposition method, the deflection equation of the fiber is obtained by Equation (7):

(7) $$z=\frac{\sigma_{b}}{k_{b}}+\frac{F_{z}}{F_{x}}\sin\sqrt{\frac{F_{x}}{E_{f}I_{f}}}x+\left(\frac{2\lambda \sigma_{b}}{k_{b}}+\frac{2\lambda^{2}\sigma_{b}}{k_{b}}-\frac{F_{z}}{F_{x}}\right)x$$

It can be seen from Equation (7) that the fiber deformation increases with the increasing of *F_x_* and *F_z_*, and reducing *F_x_* and *F_z_* can be an effective way to reduce the fiber deformation in the drilling process. As such, the fiber can be cut effectively; the burrs and delamination were avoided in the drilling process of AFRP.

## 3. Low-Damage Drill Design and Analysis

### 3.1. Design of the New Drill

**Figure 3:**
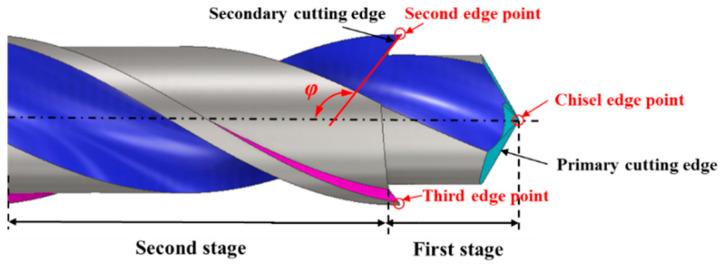
Schematic of the novel three-point step drill.

As mentioned, the low-damage drill bit was designed based on the method of reducing the *F_z_* and *F_x_*. The cutting force is mainly determined by the cutting amount of material, and drilling the material step-by-step is an effective way to reduce the *F_z_* and *F_x_*. Thus, the step structure was applied to the drill bit to reduce the damages in drilling AFRP. More importantly, as shown in Figure 2, the *F_x_* is closely related to the point angle *φ* of the drill; when *φ* > 90°, the direction of *F_x_* will be reduced, and it is beneficial for cutting fibers. Therefore, based on the above, the three-point step drill with three edge points and two steps was designed in this study, as shown in Figure 3. The first edge point is the chisel edge point in the first stage, and two edge points for the secondary cutting are in the second stage.

### 3.2. Drilling Mechanism of the Three-Point Step Drill

**Figure 4:**
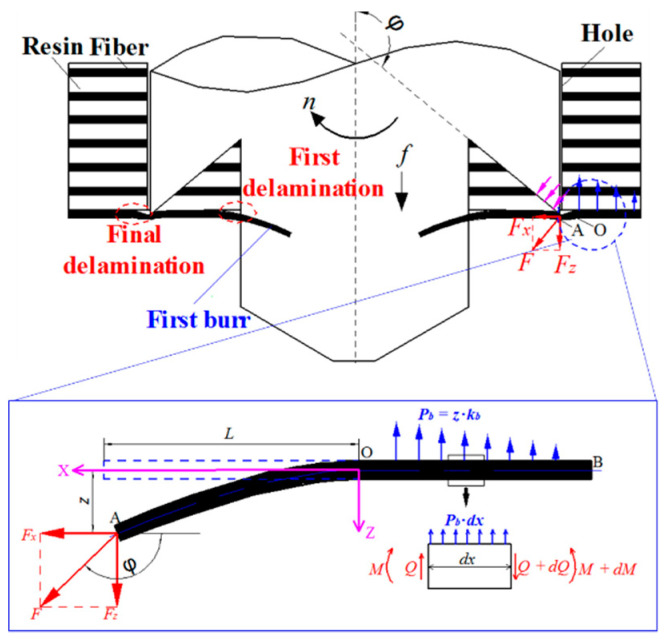
Drilling processes of the second stage of the three-point step drill.

As shown in Figure 4, the novel three-point step drill consists of functional parts that are the chisel edge point, the primary cutting edge, the second and third edge points and the secondary cutting edge. In the drilling process, the chisel edge point firstly drills out the AFRP, and the primary cutting edge drills the AFRP, forming the pilot hole; while the drill is fed through, the second and third edge points connect with the AFRP, and the secondary cutting edge removes the AFRP again, forming the final hole. Then, the drill-exit process of the second and third edge points is the key for final quality, which is shown in Figure 4. Based on Section 2.2, applying a similar model, the deformation and deflection of fiber at point O are shown in Equation (8):

(8) $$\left\{ \begin{array}{l} {z_{2}|}_{x=0}=\frac{\sigma_{b}}{k_{b}}\\ {\theta_{2}|}_{x=0}=\frac{2\lambda \sigma_{b}}{k_{b}}+\frac{2\lambda^{2}\sigma_{b}}{k_{b}} \end{array}\right.$$

The deflection equation of the fiber on the right side of the O can be obtained as:

(9) $$E_{f}I_{f}\frac{d^{2}z_{1}}{dx^{2}}=-F_{z}\cdot x+z_{1}\cdot F_{x}\;$$

Combing the boundary conditions and the displacement superposition method, the deflection equation of the fiber is obtained in Equation (10):

(10) $$z=\frac{\sigma_{b}}{k_{b}}+\frac{F_{z}}{2F_{x}}\sqrt{\frac{E_{f}I_{f}}{F_{x}}}\left(e^{-\sqrt{\frac{F_{x}}{E_{f}I_{f}}}x}-e^{\sqrt{\frac{F_{x}}{E_{f}I_{f}}}x}\right)+\left(\frac{F_{z}}{F_{x}}+\frac{2\lambda \sigma_{b}}{k_{b}}+\frac{2\lambda^{2}\sigma_{b}}{k_{b}}\right)x$$

As shown in Equations (4) and (9), the torque on the fiber from the cutting edge of the novel three-point step drill is less than that of a traditional twist drill. Additionally, comparing Equation (10) with Equation (7), the fiber deflection is also suppressed under this drilling condition of a novel three-point step drill. As the result, the fiber is cut effectively and the drilling damages can be reduced.

## 4. Experimental Verification

To obtain the influences of a three-point step drill on the surface damages’ reduction for drilling AFRP, experiments were carried out with a twist drill, a candle core drill and a three-point step drill. By observing the drilling process and drilling damages (delamination and burrs), and quantitatively characterizing and summarizing the quality, the quality improvement of AFRP when using the three-point step drill was obtained.

### 4.1. Experimental Details

**Figure 5:**
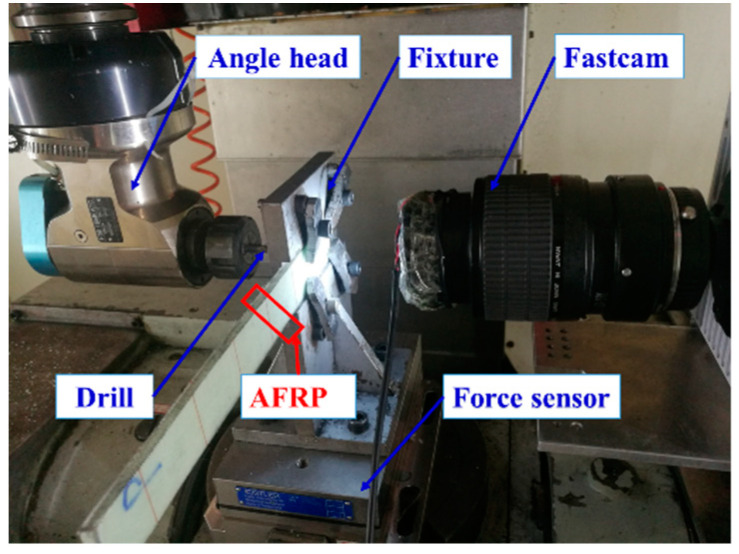
Experiment layout.

The experiment was carried in GONA 5-axis machining center (Kede CNC Co., Ltd., Dalian, China) for drilling under dry conditions, and the experiment layout is shown in Figure 5. The thrust forces were measured by the Kistler 9253B force measurement system at 3000 Hz (Kistler Company, Winterthur, Switzerland), and the force signal was filtered at 10 Hz. At the same time, the angle head was used to realize the 1:1 turning of the main movement direction of the machine tool, so that the Photron Fastcam Sa5 high-speed camera (Photron Company, Tokyo, Japan) was used to observe the drilling process of AFRP with the image acquisition frequency of 1000 frame/s. The drill-exit quality was observed under VHX-600E Ultra (Keyence, Osaka, Japan) and Alicona Infinite Focus G5 (Alicona Company, Graz, Austria), and microscopic damage was observed under scanning electron microscope (FEI Company, OR, USA).

**Figure 6:**
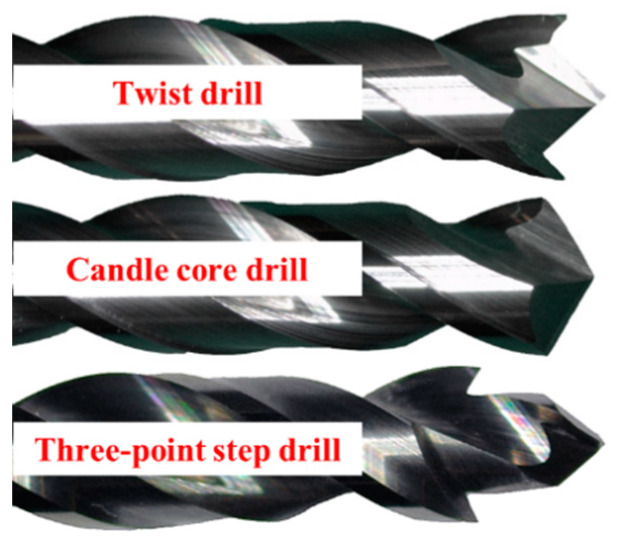
The three kinds of drills for the experiment.

The drills were manufactured by Dalian Lanqi Co., Ltd. (Dalian, China) using K44UF tungsten-cobalt carbide without a coating, as seen in Figure 6. The flute angle of the three drills was 30° and the diameter of them was 6 mm. In the experiment, the spindle speed was 3000 rpm and the feed speed was 0.02, 0.04 or 0.06 mm/rev. Each drilling experiment was repeated three times to reduce the influence of accidents. The workpiece used in the experiment was a 1414 aramid fiber-reinforced epoxy resin-based composite material that was laminated in the directions of 0° and 90°—cross-layered with 4 mm in thickness. The composite contained 60% fiber. Other properties of aramid fiber and the drilling tools are shown in Table 1.

### 4.2. Experimental Results and Discussion

#### 4.2.1. Drilling Process Results

**Figure 7:**
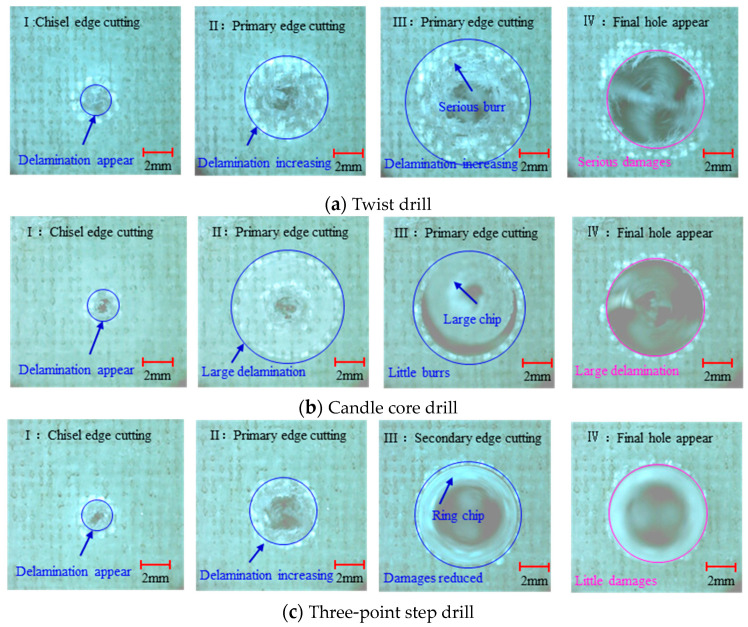
Drilling processes of three drills: (**a**) twist drill, (**b**)candle core drill, (**c**) three-point step drill.

The drilling results of edges on the drill-exit material are the key to analyzing the damages. The Photron Fastcam Sa5 high-speed photography results of the typical export stage pictures of the twist drill, candle core drill and three-point step drill with 3000 rpm speed and 0.04 mm/rev are shown in Figure 7, which shows the drilling process in four stages.

The process of twist drill drilling AFRP is shown in Figure 7a. Stage I is the stage of the chisel edge drilling out the AFRP, where the delamination appeared. With the feeding, the primary edge began to cut the material, and serious burr damage appeared at the same time in stage II and stage III. Serious damage kept the final hole formed until the primary edge was drilled out the AFRP in stage Ⅳ.

Figure 7b shows the observation results of the candle core drill in drilling AFRP. As shown, in stage I, the delamination appeared to be the same as with the twist drill. While in stage II, the delamination does not gradually expand like it does for a twist drill; a large area of delamination suddenly appears. This is caused by the primary cutting edge of the candle core drill making contact with the final hole and forcing it, forming the large chip. In stage III, the primary edge effectively cuts the AFRP from the final hole position with little burrs. Before stage IV, there was already sizable delamination damage; after the final hole was drilled, there was large delamination and small burr holes.

As for the three-point step drill—the drilling process is shown in Figure 7c—the chisel edge point drilled out the material in stage I, forming the initial delamination like the twist drill and candle core drill do. In stage II, the first step using a smaller diameter bit can allow entery, which leads to the pilot hole with delamination and burrs. After entering stage III, two second and third edge points begin to contact AFRP, but do less cutting than the candle core drill, which cuts AFRP to its diameter with less delamination and little burrs. The little-in damage hole was drilled by the three-point step drill.

**Figure 8:**
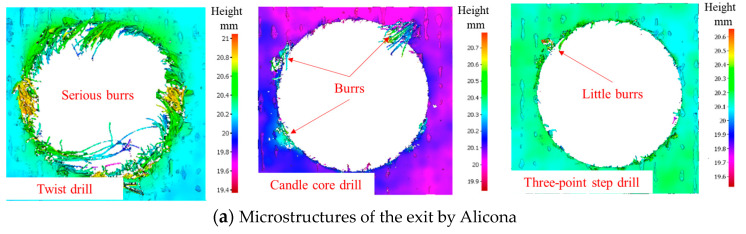

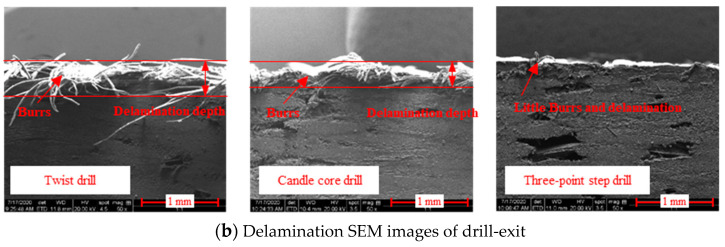
Microstructures and SEM images of the drill-exit. (**a**) Microstructures of the exit by Alicona; (**b**) Delamination SEM images of drill-exit.

As observed above, the drilling process of a three-point step drill is beneficial for improving the drilling quality, the quality of which is shown in Figure 8 by Alicona Infinite Focus G5 and SEM. It can be seen that the final hole drilled by the three-point edge drill has almost no burrs and delamination damage, and the quality is significantly better than that of the twist drill and candle core drill. Consequently, the three-point step drill has a significant effect on reducing the burrs and delamination damage of AFRP drilling.

#### 4.2.2. Quality of Drill-Exit

As above in Section 4.2.1, the drilling quality has been improved by the three-point step drill under 0.04 mm/rev, and to further verify the damage suppression effect of the three-point step drill, the drill-exit quality under different parameters is shown in Table 2. It can be seen that the delamination and burr damage of the twist drill is the most serious; the candle core has improved burr damage; and the three-point step drill has almost no burrs or delamination damage.

**Figure 9:**
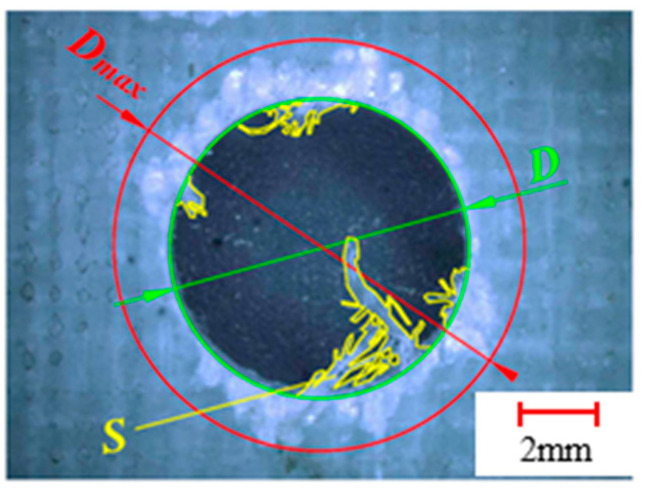
Measurement of delamination factor and burrs area.

Drilling quality is measured and compared by the burrs area and delamination factor, as shown in Figure 9. The exit burrs area *S* and the delamination damage diameter *D_max_* were obtained, and the delamination factor *F_d_* is defined as the ratio of the maximum damage diameter *D_max_* to the normal aperture *D* [36], which can be expressed as:

(11) $$F_{d}=\frac{D_{\max}}{D}$$

**Figure 10:**
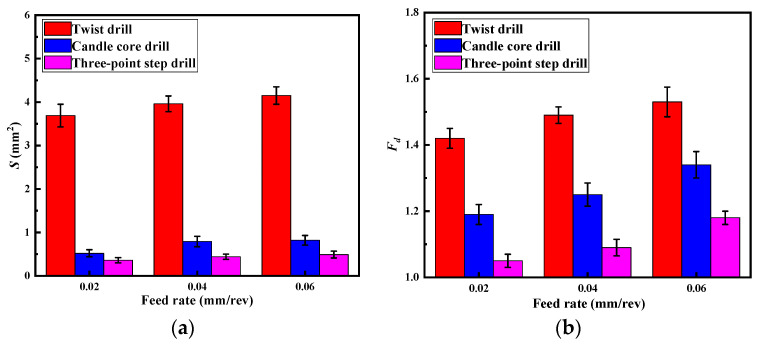
Damage variations of the three drills: (**a**) burrs area, (**b**) delamination factor.

Figure 10 illustrates the burrs area and delamination factor variations of different drills with different feeds. From Figure 10a, the burr damage caused by the three-point edge drill is only about 10% for the twist drill, but about 60% for the candle core drill. From Figure 10b, the delamination factors of the three-point edge drill were much smaller than those of the twist drill and the candle core drill. When the feed increases, the delamination factor will increase, but there is no significant change in the burrs area. This is mainly because the delamination damage is greatly affected by the thrust force, and an increase in the feed speed causes the thrust force to increase. The burr damage is mainly determined by the cutting form. The drilling forms are shown in Figure 7; the three-point step drill can cut the fiber effectively, and a change in the feed rate does not change the forms, so the burrs areas of the three-point step drill were the smallest and did not change significantly with the feed rate. Thus, 0.02 mm/rev is a suitable feed rate for the three-point step drill.

Combining the above experimental results, the three-point step drill reduced the delamination and burr damage at the same time, thereby significantly improving drilling quality of the AFRP.

#### 4.2.3. Thrust Forces

**Figure 11:**
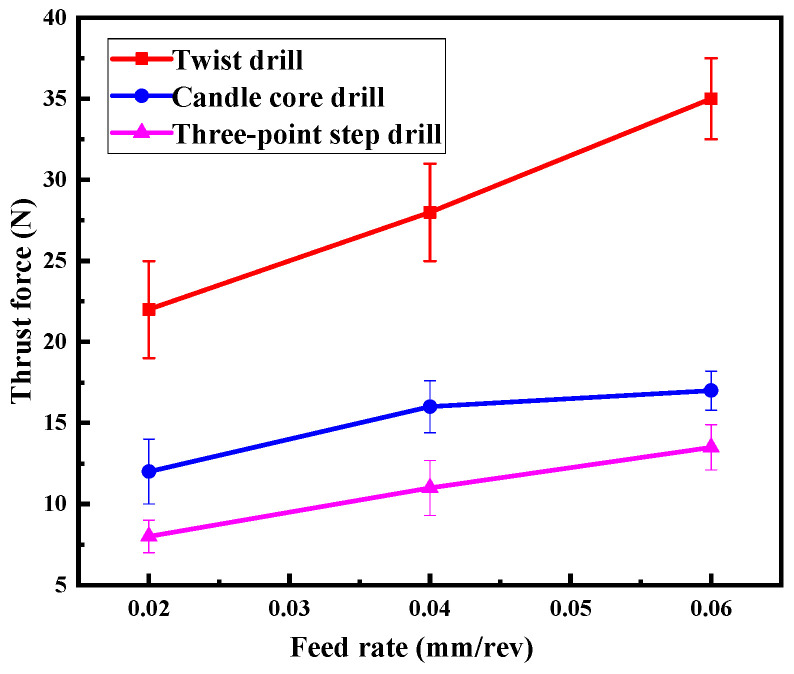
Thrust force curves of drills.

To verify the above result, the thrust force curves during the drilling process were compared in detail. Figure 11 shows the typical thrust force curves of three drills at 3000 rpm and 0.02 mm/rev. It can be seen that the thrust force kept the same trend of rising firstly and then falling. However, the thrust force of the three-point step drill was only about 36% of that of the twist drill, and about 65% of that of the candle core drill. This is because the step structure on the three-point step drill can cut the material step-by-step, which divides the thrust force into stages and reduces its value. This is the main reason for the delamination damages being reduced.

**Figure 12:**
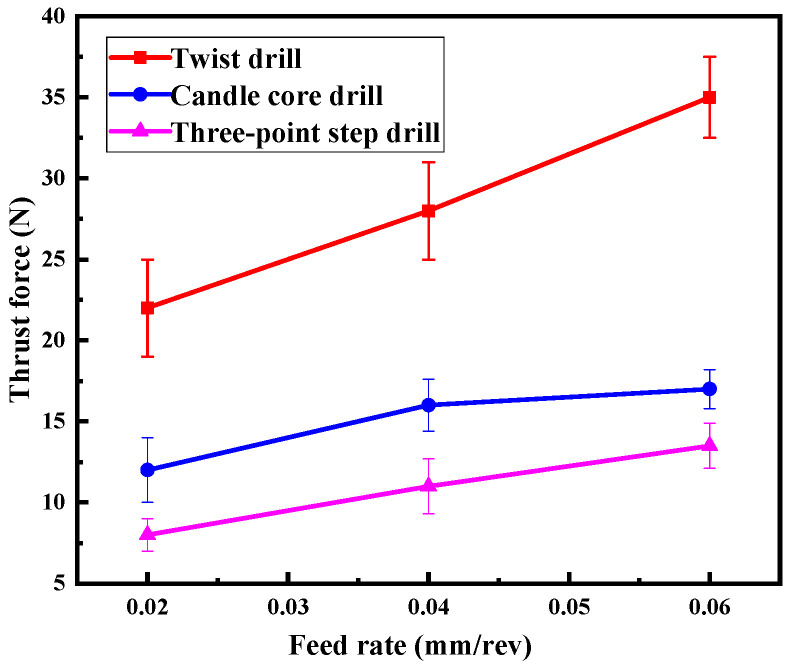
Curve of cutting force with feed rate.

At the same time, Figure 12 illustrates the thrust force variations and the feed rate. It can be seen that the thrust force increased with the increase of the feed rate for all three drills, but the thrust force of the three-point step drill was the smallest all the time. That was the reason for the increase in damages caused by the other drills seen in Figure 11. In summary, the three-point step drill significantly reduces the thrust force, leading to reduced drill damages.

## 5. Conclusions

This article proposed a low-damage method to effectively minimize the drilling damages and improve the surface quality of drilling AFRP. The cutting model of the AFRP drill-exit fiber was established and the fiber deformation was calculated. Then, the three-point step drill was proposed based on the model and the reduction in drilling damage was verified through comparative experiments. The main conclusions obtained during the research are as follows:

1. A two-dimensional cutting model of drill-exit fibers was established successfully for analyzing the fiber deformation. The thrust force and radial cutting force of the edge on the fiber have important influences on the deformation of the exit fiber, and reducing the thrust force and changing the direction of the radial cutting force on the fiber to point into the hole can effectively reduce the deflection of the exit fiber.
2. The three-point edge drill with a step structure can cut the material step-by-step, and three points can cut the aramid fiber effectively; the thrust force was reduced, leading to delamination being decreased and fewer, smaller burrs were cut by the three-point structure.
3. Comparative milling experiments’ results proved that the three-point step drill can reduce the delamination and burrs at the same time, compared to the twist drill and candle core drill. The feed rate increase led to a drilling delamination increase, but had little effect on the burr damage. The three-point step drill with a small feed rate effectively removed the burrs and avoided delamination, making it a complete low-damage drilling method for AFRP.

## Figures and Tables

**Table 1:** Parameters of drills and aramid fiber.

Drill and Material	Project	Parameter
Drills	Cutting Edge Length/mm	40
	Blade Number	15
	Diameter/mm	6
	Helix Angle/°	30
	First Stage Diameter of Three-Point Step Drill/mm	4.88
Aramid Fiber	Tensile Modulus/GPa	2.8
	Elastic Modulus/GPa	75–95
	Density/(g/cm^3^)	1.44
	Fiber Diameter/um	12
	Elongation/%	3.510–3.730

**Table 2:** Damage pictures of the outlets of the holes.

Tool	0.02 mm/rev	0.04 mm/rev	0.06 mm/rev
Twist Drill	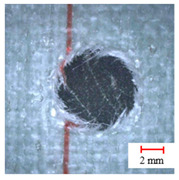	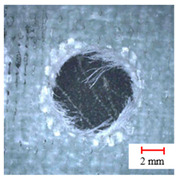	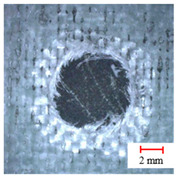
Candle Core Drill	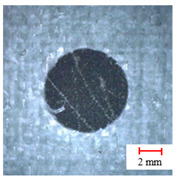	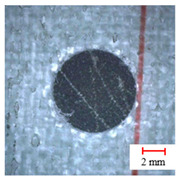	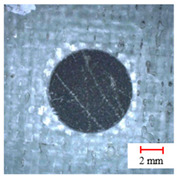
Three-Point Step Drill	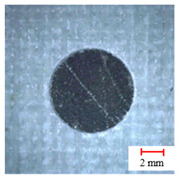	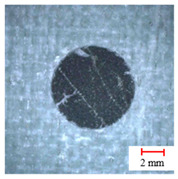	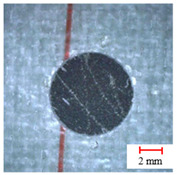

## References

[1] Li Z., Cheng X., He S., Shi X., Gong L., Li Z. (2016). Aramid fibers reinforced silica aerogel composites with low thermal conductivity and improved mechanical performance. Compos. Part A Appl. Sci. Manuf..

[2] Homae T., Shimizu T., Fukasawa K., Masamura O. (2006). Hypervelocity Planar Plate Impact Experiments of Aramid Fiber-reinforced Plastics. J. Reinf. Plast. Compos..

[3] Okhawilai M., Parnklang T., Mora P., Hiziroglu S., Rimdusit S. (2018). The energy absorption enhancement in aramid fiber-reinforced poly(benzoxazine-co-urethane) composite armors under ballistic impacts. J. Reinf. Plast. Compos..

[4] Dharan C., Won M. (2000). Machining parameters for an intelligent machining system for composite laminates. Int. J. Mach. Tools Manuf..

[5] Liu S., Yang T., Liu C., Du Y. (2019). Comprehensive investigation of cutting mechanisms and hole quality in dry drilling woven aramid fibre–reinforced plastic with typical tools. Proc. Inst. Mech. Eng. Part B J. Eng. Manuf..

[6] Masoud F., Sapuan S., Ariffin M.M., Nukman Y., Bayraktar E. (2020). Cutting Processes of Natural Fiber-Reinforced Polymer Composites. Polymers.

[7] Abrão A., Faria P., Rubio J.C., Reis P., Davim J.P. (2007). Drilling of fiber reinforced plastics: A review. J. Mater. Process. Technol..

[8] Sun D., Lemoine P., Keys D., Doyle P., Malinov S., Zhao Q., Qin X., Jin Y. (2016). Hole-making processes and their impacts on the microstructure and fatigue response of aircraft alloys. Int. J. Adv. Manuf. Technol..

[9] Nor F.M., Lim J.Y., Tamin M.N., Lee H.Y., Kurniawan D. (2020). Effects of Starter Defect on Energy Release Rate of Three-Point End-Notch Flexure Tested Unidirectional Carbon Fiber Reinforced Polymer Composite. Polymers.

[10] Zhang L.C., Zhang H.J., Wang X.M. (2001). A Force Prediction Model for Cutting Unidirectional Fibre-Reinforced Plastics. Mach. Sci. Technol..

[11] Pwu H.Y., Hocheng H. (1998). Chip Formation Model of Cutting Fiber-Reinforced Plastics Perpendicular to Fiber Axis. J. Manuf. Sci. Eng..

[12] Xu W., Zhang L. (2016). Mechanics of fibre deformation and fracture in vibration-assisted cutting of unidirectional fibre-reinforced polymer composites. Int. J. Mach. Tools Manuf..

[13] Li H., Qin X., He G., Price M.S.T., Jin Y., Sun D. (2017). An energy based force prediction method for UD-CFRP orthogonal machining. Compos. Struct..

[14] Jia Z., Fu R., Niu B., Qian B., Bai Y., Wang F. (2016). Novel drill structure for damage reduction in drilling CFRP composites. Int. J. Mach. Tools Manuf..

[15] Su F., Zheng L., Sun F., Wang Z., Deng Z., Qiu X. (2018). Novel drill bit based on the step-control scheme for reducing the CFRP delamination. J. Mater. Process. Technol..

[16] Kim S.-C., Kim J.-S., Yoon H.-J. (2011). Experimental and numerical investigations of mode I delamination behaviors of woven fabric composites with carbon, Kevlar and their hybrid fibers. Int. J. Precis. Eng. Manuf..

[17] Wan Y., Chen G., Huang Y., Li Q., Zhou F., Xin J., Wang Y. (2005). Characterization of three-dimensional braided carbon/Kevlar hybrid composites for orthopedic usage. Mater. Sci. Eng. A.

[18] Bunsell A.R. (1975). The tensile and fatigue behaviour of Kevlar-49 (PRD-49) fibre. J. Mater. Sci..

[19] Wang F., Yan J.-B., Zhao M., Wang D., Wang X.-N., Hao J.-X. (2020). Surface damage reduction of dry milling carbon fiber reinforced plastic/polymer using left–right edge milling tool. J. Reinf. Plast. Compos..

[20] Biermann D., Bathe T., Rautert C. (2017). Core Drilling of Fiber Reinforced Materials using Abrasive Tools. Procedia CIRP.

[21] Wu M., Tong X., Wang H., Hua L., Chen Y. (2020). Effect of Ultrasonic Vibration on Adhesive Bonding of CFRP/Al Alloy Joints Grafted with Silane Coupling Agent. Polymers.

[22] Liu S., Yang T., Liu C., Jin Y., Sun D., Shen Y. (2020). Modelling and experimental validation on drilling delamination of aramid fiber reinforced plastic composites. Compos. Struct..

[23] Zheng X., Dong D., Huang L., Wang X., Chen M. (2013). Investigation of tool wear mechanism and tool geometry optimization in drilling of PCB fixture hole. Circuit World.

[24] Shuaib A.N., Al-Sulaiman F.A., Hamid F. (2004). Machinability of Kevlar^®^ 49 Composite Laminates While Using Standard TiN Coated HSS Drills. Mach. Sci. Technol..

[25] Veniali F., Di Ilio A., Tagliaferri V. (1995). An Experimental Study of the Drilling of Aramid Composites. J. Energy Resour. Technol..

[26] Iqbal M., Bahri S., Akram A. (2019). Effect of cutting parameter on tool wear of HSS tool in drilling of Kevlar composite panel. Proceedings of the IOP Conference Series: Materials Science and Engineering.

[27] Hao J., Wang F., Zhao M., Bai Y., Jia Z. (2020). Drill bit with clip-edges based on the force control model for reducing the CFRP damage. J. Reinf. Plast. Compos..

[28] Bishop G., Gindy N. (1990). An investigation into the drilling of ballistic Kevlar composites. Compos. Manuf..

[29] Bhattacharyya D., Horrigan D. (1998). A study of hole drilling in Kevlar composites. Compos. Sci. Technol..

[30] Lei J., Jing Q. (2019). Performance Analysis of Drilling Test of Aramid Fiber Composite. Proceedings of the IOP Conference Series: Materials Science and Engineering.

[31] Anarghya A., Harshith D., Rao N., Nayak N.S., Gurumurthy B., Abhishek V., Patil I.G.S. (2018). Thrust and torque force analysis in the drilling of aramid fibre-reinforced composite laminates using RSM and MLPNN-GA. Heliyon.

[32] Díaz-Álvarez A., Rodríguez-Millán M., Miguélez M. (2018). Experimental analysis of drilling induced damage in aramid composites. Compos. Struct..

[33] Gao H., Zhuang Y., Wang B., Huang J.L. (2012). Study on the Combined Machining Technology of Sawing and Grinding for Drilling Aramid/Epoxy Composites. Adv. Mater. Res..

[34] Liu S., Yang T., Liu C., Du Y., Gong W. (2018). Investigation of hole quality during drilling of KFRP based on the interaction between collars and cutter. Int. J. Adv. Manuf. Technol..

[35] Qi Z., Zhang K., Cheng H., Wang D., Meng Q. (2015). Microscopic mechanism based force prediction in orthogonal cutting of unidirectional CFRP. Int. J. Adv. Manuf. Technol..

[36] Xu J., Li C., Mi S., An Q., Chen M. (2018). Study of drilling-induced defects for CFRP composites using new criteria. Compos. Struct..

